# Lipid emulsion injection-induced reversal of cardiac toxicity and acceleration of emergence from general anesthesia after scalp infiltration of a local anesthetic: a case report

**DOI:** 10.1186/s40981-017-0077-6

**Published:** 2017-02-10

**Authors:** Rintaro Hoshino, Yoshinori Kamiya, Yuka Fujii, Tsunehisa Tsubokawa

**Affiliations:** 1 Division of Anesthesiology, Niigata University Graduate School of Medical and Dental Sciences, 1-757 Asahimachi-dori, Chuo Ward, Niigata, 951-8510 Japan; 2 Department of Anesthesiology, Uonuma Institute of Community Medicine, Niigata University Medical and Dental Hospital, 4132 Urasa, Minami-uonuma, Niigata, 949-7302 Japan; 3 Department of Anesthesiology, Faculty of Medicine, Institute of Medical, Pharmaceutical and Health Science, Kanazawa University, 13-1 Takara-machi, Kanazawa, Ishikawa 920-8641 Japan; 4 Department of Anesthesiology, Ishikawa Prefectural Central Hospital, 2-1 Kuratsuki-higashi, Kanazawa, Ishikawa 920-8201 Japan; 5 Department of Anesthesiology, The Jikei University School of Medicine, 3-25-8 Nishi-shinbashi, Minato Ward, Tokyo, 105-8461 Japan

**Keywords:** Local anesthetic intoxication, Delayed emergence, Brain surgery, Lipid rescue, Lipid sink theory

## Abstract

**Background:**

A scalp block or wound infiltration of local anesthetic is thought to effectively control post-craniotomy pain. However, it can result in local anesthetic toxicity (LAST), which is difficult to distinguish from brain damage due to the surgical procedure when emergence from general anesthesia is delayed. Lipid rescue (infusion of a lipid emulsion) is a widely accepted treatment for LAST.

**Case presentation:**

A 64-year-old man underwent surgical resection of a glioma in the brainstem. While still under general anesthesia, and before suturing of the wound, he received a 20-mL scalp infusion of ropivacaine 0.75%. His emergence from anesthesia was delayed, his respiration was suppressed, and premature ventricular contractions occurred; all of which are symptoms of LAST. Injection of a 20% lipid emulsion rapidly alleviated these symptoms. Interestingly, the blood concentration of ropivacaine increased after lipid rescue.

**Conclusions:**

The increase in ropivacaine concentration in the blood after lipid rescue suggests that the intravenously administered lipid emulsion absorbed the ropivacaine from the intoxicated brain and heart tissue. This finding is consistent with the lipid sink theory as a mechanistic explanation of lipid rescue.

## Background

A scalp block or wound infiltration of local anesthetics is thought to effectively control post-craniotomy pain [1]. However, because the scalp has a rich blood flow, anesthetics locally injected into the scalp are rapidly absorbed, which increases the risk of local anesthetic toxicity (LAST) [2, 3]. It is difficult to distinguish between brain damage and LAST when emergence from general anesthesia is delayed.

Although numerous studies suggest that lipid rescue (administration of a lipid emulsion) effectively treats LAST [4–6], only a few have described LAST under general anesthesia conditions [7–9]. Reports detailing changes in the blood concentrations of local anesthetics after lipid rescue in the clinical setting are also rare [10]. Herein, we present a case in which lipid rescue effectively alleviated LAST in a patient emerging from general anesthesia after brain tumor surgery and a scalp block using the local anesthetic ropivacaine. Paradoxically, the blood concentration of ropivacaine was higher after lipid rescue compared with that before.

## Case presentation

A 62-year-old man (height and weight, 165 cm and 64.2 kg, respectively) had a glioma on the right side of his brainstem. He was alert but experienced left hemiparesis. He had untreated mild diabetes mellitus (hemoglobin A1c 6.3%), as well as mild hypertension for which he was prescribed an angiotensin II receptor antagonist (candesartan cilexetil, 4 mg once daily) and a Ca^2+^ channel blocker (amlodipine, 2 mg once daily). Surgical resection of the glioma under general anesthesia was planned, with neurological monitoring (motor and sensory evoked potentials) to determine the resectable margin of the tumor.

General anesthesia was induced via target-controlled infusion of propofol (4 μg/mL using a TERUMO infusion pump [TE-371; Terumo Corp. Ltd., Tokyo, Japan]) and continuous infusion of remifentanil (0.5 μg/kg/min), followed by administration of rocuronium (0.6 mg/kg). After tracheal intubation, anesthesia was maintained via target-controlled infusion of propofol (1.8–3.5 μg/mL) and continuous infusion of remifentanil (0.1–0.3 μg/kg/min). The bispectral index (BIS) was used to titrate the propofol dose and was maintained at 40–60 throughout the anesthesia. Fentanyl was injected intermittently for transitional analgesia (50–100 μg). The final fentanyl dose (100 μg) was administered more than 80 min before the end of the surgery, and the total fentanyl dose during the surgery was 500 μg. Neurological monitoring during the surgery was performed in the absence of a muscle relaxant.

During the surgery, there were no premature ventricular contractions (PVCs) or other arrhythmias, and the patient’s circulation and other vital signs were stable. The patient received an infusion of 1800 mL of a crystalloid solution, 1000 mL of a colloid solution (6% hydroxyethyl starch in normal saline), and 300 mL of 20% mannitol (total infusion volume 3100 mL) during the surgery. The blood loss was 865 mL, but the patient did not receive a blood transfusion.

After bony closure of the skull, 20 mL of ropivacaine 0.75% (2.34 mg/kg) was infiltrated around the wound edge. The operating time was 8 h and 42 min. At the end of the surgery, the propofol/remifentanil infusion was terminated. However, 30 min later, the patient had not regained consciousness or spontaneous breathing, even though the estimated blood concentrations of propofol and remifentanil were below subanesthetic levels. At that time, the BIS was approximately 70. The patient did not become hypothermic during surgery, and his core body temperature was above 37°C at the end of the procedure. Blood gas analysis revealed that the blood pH, oxygenation status, ventilation status, and electrolyte status were within normal levels (pH, 7.345; PaCO_2_, 40.7 torr; PaO_2_, 129.2 torr; HCO_3_^–^, 21.7 mEq/L; base excess, -3.7; Na^+^, 140.6 mEq/L; K^+^, 3.58 mEq/L; and Ca^2+^, 1.168 mg/dL) under mandatory ventilation (F_IO2_, 0.4; V_T_, 500 mL; respiratory rate, 10/min). However, the PVC rate gradually increased to 15 beats/min. Taken together, these observations led us to suspect that LAST had occurred 40 min after ropivacaine infiltration and 30 min after propofol/remifentanil discontinuation.

We administered a 20% lipid emulsion (Intralipos®; Ohtsuka Pharmaceuticals, Inc., Tokushima, Japan) as a bolus of 150 mL, followed by continuous infusion at a rate of 10 mL/min (total volume 250 mL). After the bolus was administered, the PVCs disappeared, the blood pressure increased from 112/62 to 162/79 mmHg, the PR interval on ECG decreased from 0.16 to 0.12 s, and spontaneous breathing resumed. After the entire dose of emulsion was administered, the patient regained consciousness and responded to call stimuli, and the tracheal tube was removed. He was observed for 30 min in the operating room and then transferred to the post-anesthesia care unit in the neurosurgical ward.

**Fig. 1:**
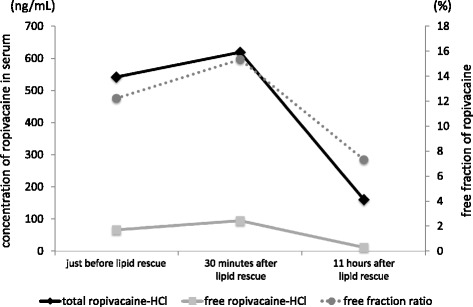
Total and free concentrations of ropivacaine just before, 30 min, and 11 h after lipid rescue. The concentrations at these time points were 540.6, 617.7, and 159.9 ng/mL (total) and 66, 94.5, and 11.6 ng/nL, respectively (free). The free fraction ratio (free/total) of ropivacaine increased from 12.2 before lipid rescue to 15.3 after lipid rescue

To determine the total and free (protein-unbound) concentrations of ropivacaine, we collected 10 mL blood samples from the arterial line just before administering the lipid emulsion, 15 min after extubation (30 min after administering the lipid emulsion), and 11 h after administering the lipid emulsion. After sampling, the blood samples were centrifuged at 5000*g* for 5 min, and the supernatant (plasma) was stored at −80 °C until analysis. The total and free ropivacaine concentrations were determined via liquid chromatography/mass spectrometry according to previously reported methods [11, 12]. Both the total and free concentrations, and the free fraction ratio of ropivacaine, were higher in 30 min after lipid rescue than before lipid rescue (Fig. 1).

## Conclusions

This report describes a case of delayed emergence from anesthesia in a patient who received a scalp infiltration of ropivacaine at the end of brain surgery. After infiltration, the frequency of ventricular arrhythmias increased and respiration was suppressed. The administration of a 20% lipid emulsion mitigated these symptoms. Owing to the timing, we thought that LAST had occurred because delayed emergence from general anesthesia and the symptoms described above were seen even though the estimated target concentrations of propofol and opioids were presumably below subanesthetic levels.

Distinguishing between symptoms of postoperative LAST and brain damage is difficult because both may induce disturbance of consciousness, and delayed emergence from general anesthesia after a craniotomy is not unusual. However, the American Society of Regional Anesthesia (ASRA) Practice Advisory on Local Anesthetic Systemic Toxicity has stated that “The practitioner’s vigilance is of critical importance in recognizing early signs of LAST (CNS depression [coma, respiratory arrest] and cardiac excitation [PVCs and tachyarrythmias]), appreciating their variable presentation, and having a low threshold for considering LAST in patients that have received potentially toxic doses of local anesthetics and manifest atypical or unexpected signs and symptoms” [13]. Delayed emergence from general anesthesia, which was one of the symptoms of LAST in a previous study [9], may have been the cause of LAST in our case because the PVC rate increased during the observation period. Administration of a lipid emulsion resulted in the disappearance of the PVCs and the return of spontaneous breathing and consciousness.

Lipid rescue was first described by Weinberg et al. [6] in 1998. They showed that an intravenously administered lipid emulsion prevented cardiac arrest and increased the likelihood of resuscitation after a cardiac arrest induced by LAST. The ASRA currently recommends lipid rescue as a first-line therapy for LAST [14]. There are several theories as to how lipid rescue reverses LAST. The well-documented, experimentally tested lipid sink theory [6, 15] suggests that the lipid emulsion absorbs the local anesthetic from several tissues (e.g., myocardium, brain, and vascular smooth muscle), which increases the metabolic capacity of the liver and, consequently, the catabolism of the local anesthetic [16].

In our case, the blood ropivacaine concentrations were higher 30 min after administration of the emulsion when compared with before. This phenomenon is strange because “free” local anesthetics are thought to have unfavorable effects including CNS and cardiac toxicity, and increases in their amounts after lipid rescue appear to worsen LAST. However, if the intravenously injected lipid emulsion absorbs the local anesthetics from the intoxicated tissue, their concentrations in the blood would transiently increase. Such an increase was observed in our study and is consistent with the lipid sink theory.

Other mechanisms may account for the lipid rescue phenomenon in patients with LAST. A recent study showed that infusion of a lipid emulsion reduced the peak concentrations of local anesthetics by 26 to 30%, presumably by expediting their clearance [17]. The lipids may also serve as an energy source for mitochondria, thereby facilitating the restoration of cellular function in intoxicated tissues [6, 18]. Alternatively, the lipids may directly promote the recovery of sodium channel function [19].

In our patient, the dose of ropivacaine was less than 3 mg/kg, and the plasma concentration of ropivacaine when LAST was suspected was less than that in a previous report [2]. In a previous study, the maximum tolerated plasma concentrations of ropivacaine in the arterial blood of healthy volunteers were shown to be in the microgram per milliliter range [20]. However, our patient had an adverse cardiac reaction that included an increase in PVC frequency even though the plasma concentration of ropivacaine was below the microgram per milliliter range. A previous study reported that intravenous administration of a small dose of ropivacaine (36.9 ± 8.6) caused cardiac conduction disturbance (elongation of the PR interval, QRS width, and QT length) and CNS symptoms in healthy male volunteers [21], and the dose of ropivacaine in that study was less than half of the maximal tolerated dose in the study by Knudsen et al. [20]. Taken together, even a small dose of local anesthetic, which has been thought not to cause adverse effects, could potentially lead to CNS and cardiovascular symptoms. In our case, the PVCs disappeared and hypotension was alleviated after infusion of the lipid emulsion. These findings suggest that the PVCs were due to LAST.

The delayed emergence from anesthesia and accompanying PVCs in our case were unexpected because we had not encountered these phenomena in previous craniotomies, even when the wound was infiltrated with 20 mL of 0.75% ropivacaine. However, previous studies on healthy volunteers suggest that relatively small doses of local anesthetics can potentially cause CNS dysfunction [20, 21]. Thereafter, we recommend that neurosurgeons use ropivacaine or levobupivacaine at concentrations less than 0.75% (e.g., 0.375 to 0.5%) for wound infiltration.

## Conclusions

We strongly recommend that LAST be considered in cases in which emergence from general anesthesia is delayed after a scalp block because overlooking this condition can result in circulatory collapse. On the basis of our own experience and previous reports, we suggest that lipid rescue is an effective and safe treatment for LAST. Lipid emulsions should be readily available in the operating room for timely treatment of LAST.

## Abbreviations

ASRA: American Society of Regional Anesthesia
BIS: Bispectral index
LAST: Local anesthetic toxicity
PVC: Premature ventricular contraction
